# Ero1-Mediated Reoxidation of Protein Disulfide Isomerase Accelerates the Folding of Cone Snail Toxins

**DOI:** 10.3390/ijms19113418

**Published:** 2018-10-31

**Authors:** Henrik O’Brien, Shingo Kanemura, Masaki Okumura, Robert P. Baskin, Pradip K. Bandyopadhyay, Baldomero M. Olivera, Lars Ellgaard, Kenji Inaba, Helena Safavi-Hemami

**Affiliations:** 1 Department of Biology, University of Utah, Salt Lake City, UT 84112, USA; henrikobrien@gmail.com (H.O.); rpbaskin@gmail.com (R.P.B.); pradip.bandyopadhyay@gmail.com (P.K.B.); olivera@biology.utah.edu (B.M.O.); 2 Institute of Multidisciplinary Research for Advanced Materials, Tohoku University, Aoba-ku, Sendai 980-8577, Japan; shingok@mail.tagen.tohoku.ac.jp (S.K.); okmasaki@mail.tagen.tohoku.ac.jp (M.O.); kinaba@tagen.tohoku.ac.jp (K.I.); 3 Department of Biology, University of Copenhagen, 2200 Copenhagen N., Denmark; lellgaard@bio.ku.dk; 4 Department of Biochemistry, University of Utah, Salt Lake City, UT 84112, USA

**Keywords:** cone snail toxins, disulfide-rich venom peptides, endoplasmic reticulum oxidoreductin-1 (Ero1), protein disulfide isomerase (PDI)

## Abstract

Disulfide-rich peptides are highly abundant in nature and their study has provided fascinating insight into protein folding, structure and function. Venomous cone snails belong to a group of organisms that express one of the largest sets of disulfide-rich peptides (conotoxins) found in nature. The diversity of structural scaffolds found for conotoxins suggests that specialized molecular adaptations have evolved to ensure their efficient folding and secretion. We recently showed that canonical protein disulfide isomerase (PDI) and a conotoxin-specific PDI (csPDI) are ubiquitously expressed in the venom gland of cone snails and play a major role in conotoxin folding. Here, we identify cone snail endoplasmic reticulum oxidoreductin-1 (*Conus* Ero1) and investigate its role in the oxidative folding of conotoxins through reoxidation of cone snail PDI and csPDI. We show that *Conus* Ero1 preferentially reoxidizes PDI over csPDI, suggesting that the reoxidation of csPDI may rely on an Ero1-independent molecular pathway. Despite the preferential reoxidation of PDI over csPDI, the combinatorial effect of Ero1 and csPDI provides higher folding yields than Ero1 and PDI. We further demonstrate that the highest in vitro folding rates of two model conotoxins are achieved when all three enzymes are present, indicating that these enzymes may act synergistically. Our findings provide new insight into the generation of one of the most diverse classes of disulfide-rich peptides and may improve current in vitro approaches for the production of venom peptides for pharmacological studies.

## 1. Introduction

Formation of correct disulfide bonds is essential for structural stability and functional integrity of most secreted proteins and peptides. Venom peptides represent one of the largest groups of small, disulfide-rich peptides found in nature [1]. These peptides have evolved to disrupt the physiology of another animal and need to be particularly stable in the extracellular environment. Multiple disulfide bonds provide the stability and structural integrity necessary for these processes. The venoms of cone snails are particularly rich in disulfide-rich peptides (termed conotoxins) and provide ideal model systems to study the molecular processes guiding disulfide-rich peptide folding in the endoplasmic reticulum (ER) of secretory cells [2,3].

Enzymes of the protein disulfide isomerase (PDI) family are known to play a central role in the biosynthesis of conotoxins by catalyzing the oxidation of cysteines into their native disulfides and isomerizing incorrectly formed disulfides into their native connections [2,4,5]. We recently showed that, in addition to canonical PDI, the venom gland of cone snails harbors a diverse family of specialized PDIs, termed conotoxin-specific PDIs (csPDIs) that significantly increase the kinetics of oxidative folding of conotoxins [2]. The PDI and csPDI-catalyzed formation of disulfide bonds involves the transfer of disulfides from the enzyme to the conotoxin substrate. PDI and csPDI contain two redox-active sites that are reduced during this process. In order to carry out further oxidations, these enzymes must be reoxidized.

How the active sites of PDI and csPDI are reoxidized upon conotoxin folding has not been addressed but has been extensively interrogated in other systems [6,7]. The major enzyme for PDI reoxidation in the mammalian ER is endoplasmic reticulum oxidoreductin-1 (Ero1) [8]. Ero1 generates disulfide bonds de novo in conjunction with a flavin adenine dinucleotide (FAD) cofactor and transfers them to PDI [9]. Ero1 family members are highly conserved proteins that are found from yeast to human [10]. In vertebrates, a gene duplication of an ancestral Ero1 gene gave rise to two distinct isoforms, Ero1α and Ero1β, that differ in their structure and tissue distribution [10].

Here, we identify Ero1 from the venom gland of the cone snail *Conus geographus* and, using expressed and purified enzymes, we investigate the role of Ero1 in the reoxidation of PDI and csPDI during the refolding of conotoxins. We show that Ero1 preferentially reoxidizes PDI over csPDI but that the folding of conotoxins is more efficient in the presence of Ero1 and csPDI over Ero1 and PDI. We further demonstrate that the highest folding rates are achieved in the presence of all three enzymes in a synergistic manner. Our findings suggest that PDI and csPDI may act synergistically during the in vivo folding of conotoxins and/or that csPDI may serve as an Ero1-independent disulfide isomerase during the oxidative folding of conotoxins.

## 2. Results

### 2.1. cDNA Sequencing of Ero1 from the Venom Gland of C. Geographus

PCR sequencing revealed the presence of two distinct Ero1 sequences that were named *Conus* Ero1 X1 and X2 based on the naming scheme used for other Ero1-like sequences from mollusks. The open reading frames (ORFs) of the two cDNA transcripts are 1362 and 1383 nucleotides long, encoding proteins of 454 and 461 amino acids in length, respectively. Full-length cDNA and predicted protein sequences have been deposited into the Genbank sequence repository (accession numbers: *Ero1_variant_X1: MK070904* and *Ero1_variant_X2: MK070905*).

**Figure 1:**
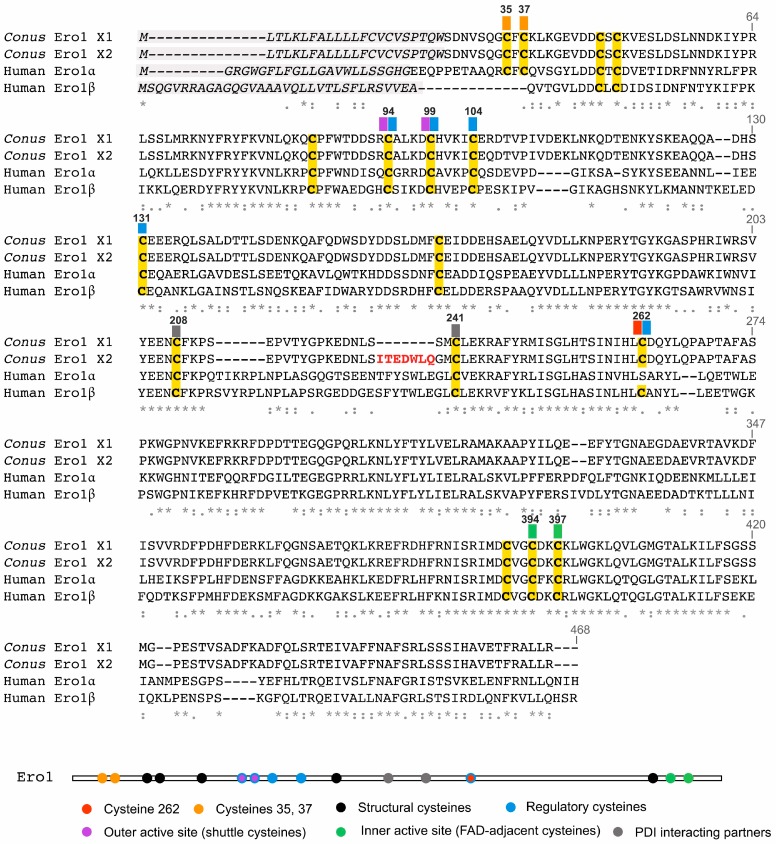
Comparative sequence alignment of the two endoplasmic reticulum oxidoreductin-1 (Ero1) isoforms identified in *Conus geographus* (*Conus* Ero1 X1 and X2) with human Ero1α and Ero1β. Predicted signal sequences are shown in gray, cysteines are highlighted in yellow. *Conus* Ero1 X1 and Ero1 X2 differ by seven amino acids (shown in red). Boxes above cysteines are color-coded according to their proposed functions [11,12,13,14]. The same color scheme is used in the schematic below the alignment. Numbering of cysteines is based on the human Ero1α sequence. Sequence alignment was generated using Multiple Alignment using Fast Fourier Transform (MAFFT)x [15]. Amino acid conservations are denoted by an asterisk (*). Full stops (**.**) and colons (**:**) represent a low and high degree of similarity, respectively. FAD = flavin adenine dinucleotide; PDI = protein disulfide isomerase.

The two predicted Ero1 isoforms only differ by seven amino acids (Figure 1) and share high amino acid sequence homology to an Ero1 sequence previously identified in the marine mollusk *Aplysia californica* (GenBank: XP_012937709, Figure S1). Consistent with this observation, phylogenetic analysis places both *Conus* enzymes well within molluskan Ero1 sequences (Figure S2). Despite the phylogenetic distance between mollusks and vertebrates, comparative alignment with human Ero1α and Ero1β reveals high sequence conservation; *Conus* Ero1 X1 and X2 share 98% identity with each other and, for example, ~48% sequence identity with human Ero1α and Ero1β. They contain the characteristic pair of shuttle cysteines that reoxidize the active site cysteines of PDI (Figure 1, purple boxes) and FAD-adjacent cysteines (Figure 1, green boxes) of other Ero1 enzymes. *Conus* Ero1 X1 and X2 contain a pair of cysteines in close proximity to the N-terminus that are present in human Ero1α but absent from Ero1β (Figure 1, orange boxes, Cys 37 and Cys 39) and also contain cysteine 262 that is characteristic for Ero1β and absent from Ero1α (Figure 1, red box). Several other residues that have previously been implicated to play a regulatory role in the human enzymes are also conserved in the *Conus* Ero1s [11,12,13,14] (Figure 1, blue boxes). Thus, *Conus* Ero1s share characteristics of both human Ero1 isoforms.

### 2.2. Conus Ero1 Expression Yields a Functional, FAD-Binding Enzyme

*Conus* Ero1 X1 (hereafter referred to as Ero1) was recombinantly expressed in *E. coli* for subsequent functional characterization. Attempts to express the other isoform, *Conus* Ero1 X2, were not successful (yields of soluble protein were <0.005 µmol/3 L of bacterial culture).

**Figure 2:**
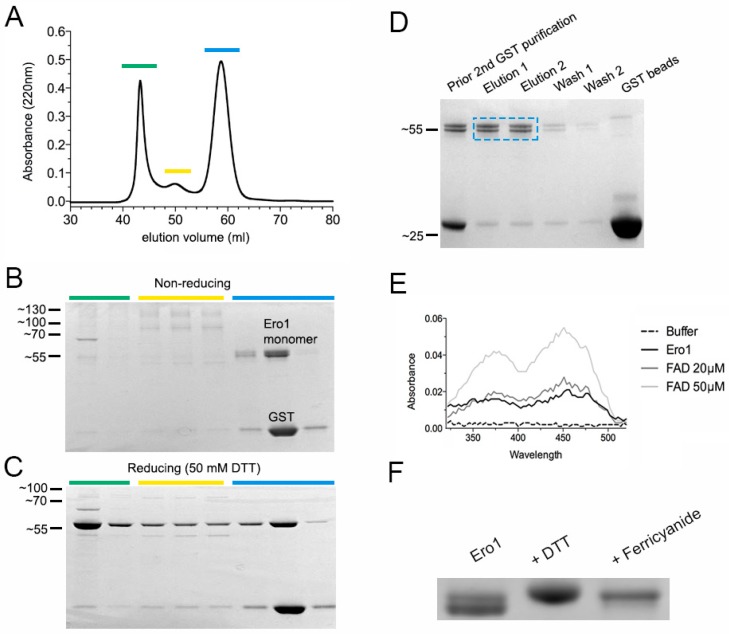
Expression and purification of recombinant *Conus geographus* Ero1 (isoform X1). (**A**) Size exclusion chromatography (SEC) chromatogram of reduced glutathione (GSH)-agarose purified Ero1 after proteolytic cleavage from the glutathione S-transferase (GST)-fusion partner. SEC fractions (colored in green, yellow and blue) were analyzed by (**B**) non-reducing and (**C**) reducing SDS-PAGE. Fractions containing the Ero1 monomer co-eluted with GST and required additional GSH-agarose purification. (**D**) SDS-PAGE of Ero1 following GSH-agarose purification. Blue box shows enzyme used for subsequent characterization. (**E**) Comparison of the absorbance spectrum of 30 µM Ero1 (in 50 mM Tris/HCl, 150 mM NaCl, pH 7.5) to that of FAD (20 and 50 µM) suggesting that each Ero1 molecule binds ~0.6 molecules of FAD. (**F**) SDS-PAGE analysis of purified Ero1 (left lane) after incubation with a reducing (dithiothreitol, DTT) and oxidizing (potassium ferricyanide) agent.

Recombinant expression of *Conus* Ero1 yielded a heterogeneous mixture of monomer and higher molecular weight species that dissociated into the monomer upon reduction with 50 mM dithiothreitol (DTT) (Figure 2A–C). Size exclusion chromatography (SEC) fractions containing the Ero1 monomer also comprised the glutathione S-transferase (GST) fusion protein that most likely eluted as a non-covalent dimer with a similar molecular weight to monomeric Ero1 (50,220 Da). Subsequent purification using reduced glutathione (GSH)-agarose yielded a final product of ~90% purity that was used for functional characterization (Figure 2D). Following elution of the GST-Ero1 construct from GSH agarose, the GST-Ero1 fusion protein appeared bright yellow, suggesting the presence of bound FAD. Spectrophotometric analysis of Ero1 following PreScission protease cleavage to release GST and purification revealed absorbance peaks at 370 and 454 nm, consistent with bound FAD at a ratio of approximately 0.6 FAD molecules/*Conus* Ero1 (Figure 2E). The final purified Ero1 monomer migrated as two distinct bands, indicating that Ero1 is expressed in two (partially) oxidized conformations. The two bands collapsed into a single, slower migrating band after treatment with either an oxidizing or reducing agent (potassium ferricyanide and DTT, respectively) (Figure 2F).

### 2.3. Conus Ero1 Preferentially Oxidizes PDI Over csPDI

**Figure 3:**
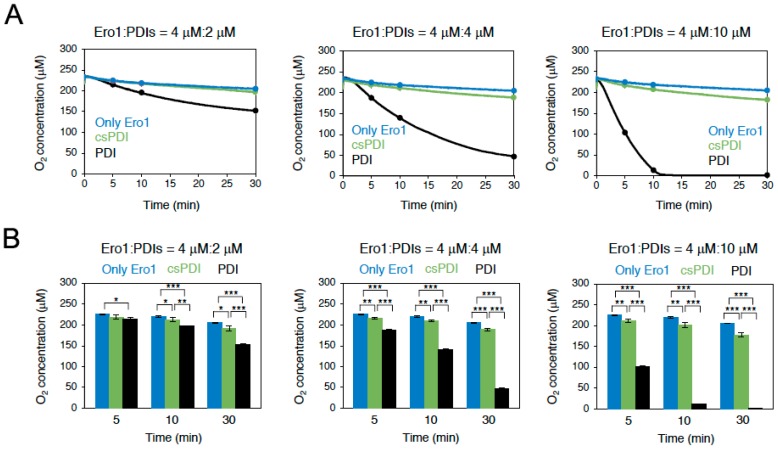
(**A**) Oxygen consumption by Ero1 (4 µM) during the oxidation of conotoxin-specific PDI (csPDI) or PDI (2, 4 or 10 µM each; left, middle and right panel, respectively) in the presence of a reducing agent (10 mM GSH). (**B**) Quantification and statistical analyses of the dissolved oxygen concentration at indicated time points in A (*n* = 3, means ± standard deviation (SD)). * *p* < 0.05; ** *p* < 0.01; *** *p* < 0.001.

Ero1 uses molecular oxygen as its preferred terminal electron acceptor; thus, oxygen consumption is a direct indicator for the rate of reoxidation of PDI in buffer containing a reducing reagent [16,17]. To investigate the reactivity of *Conus* Ero1 with PDI or csPDI, oxygen consumption during the Ero1 catalysis of PDI or csPDI oxidation was measured in the presence of GSH. Oxygen consumption assays demonstrated that Ero1 oxidized PDI with much higher efficiency than csPDI, indicating that Ero1 is highly specific to PDI (Figure 3). In line with this, Ero1-catalyzed oxidation of PDI was greatly accelerated by increasing the PDI concentration from 2 to 10 µM, whereas the rate of csPDI oxidation was little enhanced in this concentration range (Figure 3), suggesting that *Conus* Ero1 has a much higher affinity for PDI than for csPDI. Alternatively, PDI may have a much greater ability to activate *Conus* Ero1 through disulfide rearrangement than csPDI, which allows the enzyme to oxidize PDI more efficiently than csPDI [18].

### 2.4. Conus Ero1 Efficiently Oxidizes PDI but Has Low Reactivity for csPDI

**Figure 4:**
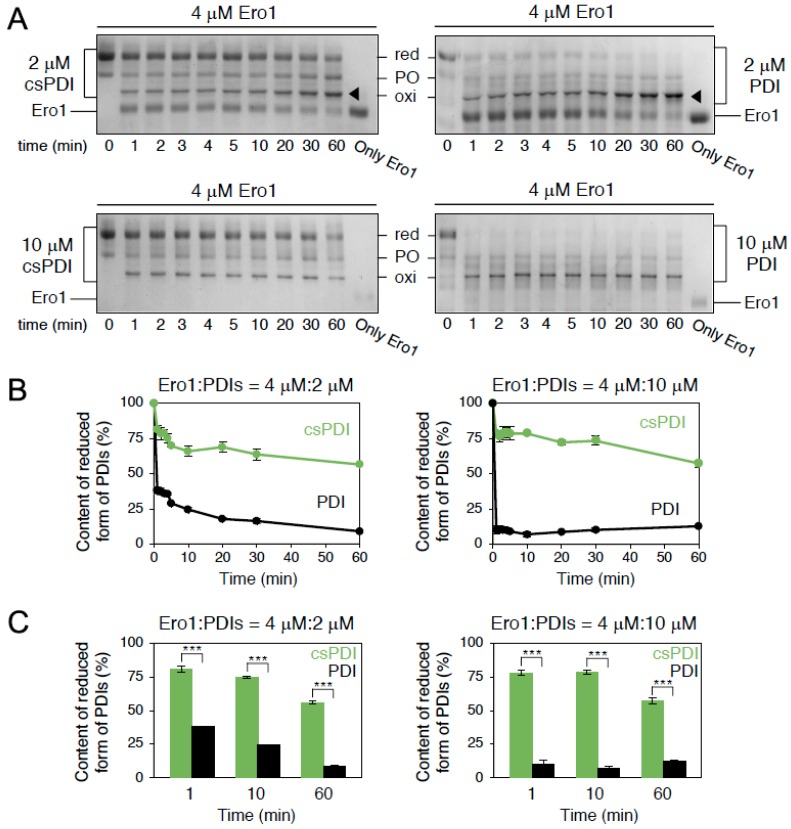
(**A**) Time course of the redox-state change of PDI or csPDI in the presence of Ero1. Experiments were performed by mixing the reduced form of PDI or csPDI (2 or 10 µM each) with Ero1 (4 µM) in air-saturated buffer at 30 °C. At the indicated time points, reactions were quenched by adding maleimide-polyethylene glycol −2k (1 mM). The redox states of PDI and csPDI were analyzed by non-reducing SDS-PAGE. red = reduced; oxi = oxidized; PO = partially oxidized; black arrow = reduced Ero1 co-migrating with oxidized PDI. (**B**) Quantification of the fraction of the reduced form of PDI or csPDI based on the gel data shown in A (*n* = 3, means ± SD). (**C**) Quantification and statistical analyses of the fraction of the reduced form of csPDI or PDI at the indicated time points in B (*n* = 3, means ± SD). *** *p* < 0.001.

Oxygen consumption assays indicated that Ero1 oxidizes PDI at faster rates than csPDI. To investigate the redox state of PDI and csPDI following Ero1 exposure, we next monitored the Ero1-catalyzed oxidation of PDI or csPDI in the absence of GSH by non-reducing SDS-PAGE. Experiments were performed with 4 µM Ero1 and 2 or 10 µM of PDI or csPDI (molar ratio of Ero1:PDI/csPDI = 1:0.5 and 1:2.5). Again, the reactivity of Ero1 for csPDI appeared low (Figure 4); csPDI largely remained in a reduced state even after 30 min, whereas PDI was almost entirely oxidized. Consistent with oxygen consumption assays, this finding suggests that Ero1 is highly specific to PDI with very little activity towards csPDI. The low reactivity of Ero1 with csPDI suggests that while the Ero1–PDI pathway is dedicated to disulfide introduction, csPDI may serve as an Ero1-independent disulfide isomerase during oxidative folding of conotoxins or that PDI and csPDI may act synergistically during the folding of conotoxin substrates.

### 2.5. Conus Ero1 Efficiently Re-Oxidizes PDI and csPDI During the Folding of Conotoxins

To determine whether *Conus* Ero1 can contribute to conotoxin folding via the reoxidation of *Conus* PDI and csPDI, folding assays of two reduced model conotoxin substrates (ω-GVIA and μ-SmIIIA) were performed in the absence and presence of 4 µM Ero1 and 2 µM PDI or csPDI. ω-GVIA and μ-SmIIIA belong to the same gene family of conotoxins (the O-superfamily), containing six cysteines that are connected via three disulfide bonds in the native peptides. ω-GVIA, from the venom of *C. geographus*, folds rapidly in vitro with very little misfolded byproduct [19]. In contrast, the in vitro oxidative folding of μ-SmIIIA, a peptide from the venom of *Conus stercusmuscarum*, is characterized by faster kinetics than ω-GVIA but significantly lower final yields [20], thus providing two substrates with well-characterized but distinct folding characteristics. Two other venom peptides from *C. geographus* have been used as model folding substrates in previous studies but were not chosen; μ-GIIIA has very similar overall folding kinetics to ω-GVIA [2,20], and α-GI has been listed as a dangerous agent by the US Centers for Disease Control and Prevention [21].

**Figure 5:**
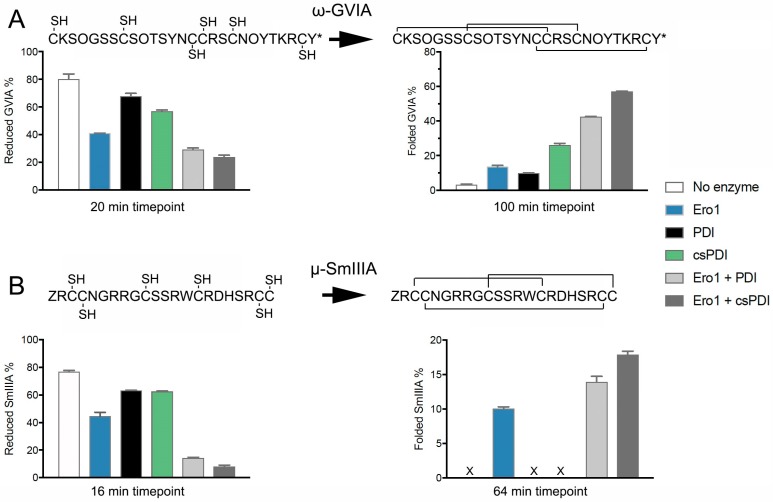
Oxidative folding assays of conotoxin (**A**) ω-GVIA and (**B**) μ-SmIIIA in the absence and presence of Ero1 and PDI or csPDI. The disappearance of the reduced form (oxidation reaction) and the appearance of the fully folded form (oxidation and isomerization reaction) are shown on the left and right, respectively. Folding assays were carried out in the absence or presence of 2 µM PDI or 2 µM csPDI and 4 µM Ero1 in 100 mM Tris-HCl, 1 mM ethylenediaminetetraacetic acid EDTA, 0.2 mM FAD, pH 7.5. Folding was initiated by adding 20 µM reduced toxin, which was quenched at different time points with formic acid and analyzed by reversed-phase chromatography. The area under the curve was measured for the reduced and fully folded forms. All reactions were carried out in duplicate. Plotted values represent mean ± SD. Toxin sequences with their native disulfide connectivity are shown above the graphs. Amino acid code: Z = pyroglutamate, O = hydroxyproline, * = C-terminal amidation. Values for statistical analyses are provided in Table S2.

The disappearance of reduced substrate (cysteine oxidation) at early time points of folding (20 and 16 min after the reaction initiation for ω-GVIA and μ-SmIIIA, respectively) and the appearance of fully folded substrate (oxidation and potential re-shuffling) at the end time point of folding (100 min and 64 min after the reaction initiation for ω-GVIA and μ-SmIIIA, respectively) were observed and quantified. PDI or csPDI alone enabled early oxidation of ω-GVIA and μ-SmIIIA as indicated by the disappearance of the reduced form and were able to catalyze the formation of the fully folded peptide for ω-GVIA, but not for μ-SmIIIA (Figure 5). Consistent with previous findings [2], csPDI was more efficient in the folding of ω-GVIA when compared to PDI. In the absence of Ero1 and/or GSH/oxidized glutathione (GSSG), some reoxidation of csPDI and PDI likely occurs in the folding buffer as previously reported [2]. Notably, Ero1 alone affected both the disappearance of the reduced toxin and the appearance of the fully folded form of μ-SmIIIA and, to a lower extent, also that of ω-GVIA. Addition of Ero1 to PDI and csPDI significantly increased the rate of disappearance of the reduced form for both enzymes (*p* < 0.005, Figure 5A,B, left panels). Similarly, addition of Ero1 to PDI and csPDI led to significantly higher yields of fully folded ω-GVIA and μ-SmIIIA at the end point of folding (*p* < 0.005, Figure 5A,B, right panels), with more efficient folding observed for substrates incubated with csPDI and Ero1 over PDI and Ero1. Values for statistical analyses are provided in Table S1.

**Figure 6:**
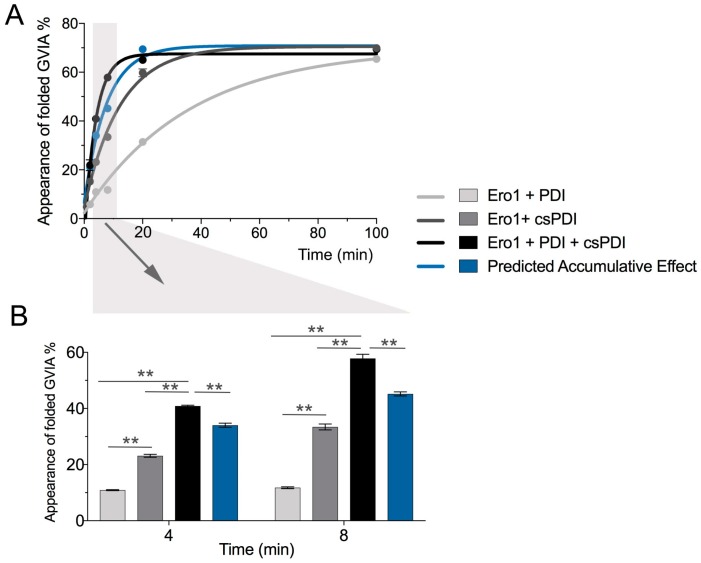
Synergistic effect of PDI and csPDI during Ero1-mediated folding of conotoxin ω-GVIA. (**A**) Folding assays were carried out in the presence of 10 µM PDI and/or 10 µM csPDI and 4 µM Ero1, quenched at different time points and analyzed by reversed-phase chromatography. The appearance of fully folded ω-GVIA was measured by taking the area under the curve of duplicate reactions (mean ± SD). (**B**) Plotted values represent folding yields obtained from early time points of folding (arrow and area shaded in gray in panel A). At 4 min and 8 min, PDI and csPDI exhibited a synergistic effect that was significantly higher than the predicted accumulative effect for both enzymes (shown in blue). Statistical analysis was performed using two-way ANOVA with time and treatment as independent variables using GraphPad Prism software (version 7). ** *p* < 0.01.

To determine a potential synergistic effect of PDI and csPDI during Ero1-mediated folding, assays were performed in the presence of Ero1 with either PDI, csPDI or both PDI and csPDI. Initial experiments using a ratio of 2:1 for Ero1 and PDI/csPDI (4 and 2 µM) showed no synergy between PDI and csPDI (data not shown). However, when the concentration of PDI/csPDI was changed to 10 µM (ratio of 1:2.5), the effect of these two enzymes in combination with Ero1 was significantly higher than the calculated cumulative effect (Figure 6, *p* < 0.005).

## 3. Discussion

The venoms of the approximately 800 species of predatory marine cone snails provide one of the most diverse sources of disulfide-rich secretory peptides in nature. Toxin expression and folding takes place in the ER of venom gland cells, where, at any given time, hundreds of distinct cysteine-rich peptides are properly folded and secreted in preparation for envenomation [3]. While a small subset of these peptides contains domains that are widely distributed in the animal and plant kingdom (e.g., the inhibitor cystine knot [22], kunitz-type domains [23], and the insulin/relaxin-like fold [24]), a large fraction of conotoxins display unique structural domains, expressed only in cone snail venom. Understanding how these structural scaffolds are efficiently folded is likely to reveal general insights into the molecular mechanisms of peptide folding. As the proper folding of disulfide-containing peptides is critical for their biological activity in prey and predator species, the enzymes involved in these processes play an important role in fitness and survival. PDI is one of the most highly expressed and abundant soluble enzymes in the venom gland of cone snails, and we recently showed that the venom gland also harbors a large and diverse gene family of PDI (csPDI) that originated by gene duplication from an ancestral PDI gene [2]. Both enzymes appear to play a role in conotoxin folding [1,2,4,25,26], but the folding of several model conotoxin substrates is more efficient in the presence of csPDI over PDI [2]. In vitro folding reactions are typically carried out in the presence of a mixture of reduced and oxidized glutathione (GSH/GSSG), molecules that were long thought to be paramount for ER-mediated protein folding. However, the discovery of several enzyme-assisted pathways, including the reoxidation of PDI by the enzyme Ero1 [27,28], dramatically changed this paradigm.

How members of cone snail PDI and csPDI are reoxidized in vivo had not been addressed, but recent advances in metagenomics sequencing now allows for the interrogation of candidate enzymes in the *Conus* venom gland that are known to play a role in other biological systems. Here, we sequenced, expressed and functionally characterized Ero1 from the venom gland of the cone snail *C. geographus*. Sequences of two different isoforms of Ero1 could be retrieved from *C. geographus*. Both showed high sequence homology to other molluskan Ero1-like sequences and only differ from each other by a short insertion/deletion (indel) of seven amino acids in a flexible loop located between Cys208 and Cys241 (numbering according to human Ero1α) [14]. Notably, mining of the GenBank protein database revealed that several other mollusks express Ero1-like sequences that only differ by having indels in this region (e.g., four and seven different putative isoforms were retrieved from *Aplysia californica* and *Biomphalaria glabrata*, respectively; Figure S1).

To the best of our knowledge, Ero1-like enzymes from invertebrates have not yet been functionally characterized, but recent studies on human Ero1α suggest that the Cys208–Cys241 pair interacts with PDI to fine-tune Ero1 activity [12,14]. Thus, the presence of different isoforms of Ero1 with indels in this flexible loop region suggests potential differences in the regulatory activity between these isoforms. Attempts to functionally express both isoforms sequenced from *C. geographus* failed due to very low yields of soluble, monomeric enzyme for the *C. geographus* Ero1 isoform X2 (the isoform containing seven additional residues between Cys208–Cys241). Constructs and conditions used for Ero1 expression and purification were identical between the two isoforms, suggesting that the low yield of monomeric Ero1 for isoform X2 was a result of amino acid differences in the Cys208–Cys241 loop region. Future studies are needed to shed light on the role of this region for *Conus* Ero1 and other molluskan Ero1-like sequences.

Recombinant expression of *Conus* Ero1 (isoform X1) provided monomeric as well as dimeric and oligomeric protein species that collapsed into the monomeric form upon treatment with a disulfide reducing agent. This is consistent with observations on the bacterial expression of human Ero1 [11,27]. Here, only the monomeric fraction was used for subsequent functional studies.

When tested in oxygen consumption assays in the presence of *Conus* PDI or csPDI, depletion of oxygen was highest in the presence of PDI and nearly no oxygen was consumed when csPDI was present. Gel shift assays confirmed that *Conus* Ero1 is indeed highly specific for PDI and has very little oxidation activity towards csPDI. These observations suggest that Ero1 may have a much lower binding affinity for csPDI compared to PDI, or that their interaction with Ero1 is driven by differences in the redox state between csPDI and PDI. While the redox state of *Conus* PDI and csPDI remains to be determined, we previously noted a conspicuous difference in the +2 position C-terminus of the CGHC active site motif in both thioredoxin-like catalytic domains (CGHCKK in the catalytic a and a′ domains in PDI and CGHCKA and CGHCKQ in the a and a′ domains of csPDI, respectively) [2]. We are not aware of any systematic investigation of the potential functional consequence of mutating residues at this position, but the close proximity to the active site could well indicate an influence of residues at this position in modulating the active-site reduction potential and thereby the redox activity of these enzymes. Future mutational studies at the +2 position C-terminus of the active site of PDI and csPDI are likely to shed light on how these changes may affect the redox state of these enzymes and their interaction with Ero1.

Structure–function studies of the interaction between human Ero1 and PDI have shown that disulfide exchange is facilitated by the binding of a protruding β-hairpin loop of Ero1 to a hydrophobic pocket in the b′ domain of PDI [11,29,30]. Homology modeling of *Conus* PDI and csPDI based on the crystal structure of human PDI previously demonstrated that while the hydrophobic patch in the b′ domain of PDI and csPDI remains largely conserved, there is a high degree of variation in the individual amino acids in the b′ domain, including the hydrophobic pocket [31]. Thus, structural differences in the b′ domain between PDI and csPDI may result in low binding affinity between *Conus* Ero1 and csPDI, which in turn leads to low reoxidation rates when compared to PDI.

The low reactivity of Ero1 with csPDI suggests that while the Ero1–PDI pathway is dedicated to disulfide introduction, csPDI may serve as an Ero1-independent disulfide isomerase during the oxidative folding of conotoxins. Oxidative folding assays using two model conotoxin substrates demonstrated that, despite the low reactivity of Ero1 with csPDI, the combination of these two enzymes resulted in more efficient folding of the two toxin substrates when compared to the combined effect of Ero1 and PDI. While csPDI is known to be a more effective foldase of conotoxins than PDI in buffer systems containing GSH/GSSG [2], the present finding of Ero1-assisted conotoxin refolding in the absence of GSH/GSSG (but presence of Ero1) was surprising, given that Ero1 had nearly no effect on the reoxidation of csPDI. Notably, Ero1 alone (without PDI or csPDI) resulted in the oxidation of both toxin substrates, as observed by the disappearance of the reduced form from the folding mixture and the appearance of fully folded toxin. One possible explanation that warrants further investigation could be that Ero1 oxidizes and csPDI isomerizes the peptide.

However, the refolding of both toxins was significantly more efficient in the presence of PDI and csPDI, with the highest folding yields achieved when all three enzymes were present. In all cases, the combinatorial effect was higher than the cumulative effect, suggesting that these enzymes act synergistically. The cooperative interaction between different members of the PDI family was previously shown for human PDI and the PDI family member P5 during peroxiredoxin-4 mediated refolding of RNase A [32].

We are not aware of any studies suggesting that Ero1 directly transfers oxidizing equivalents to PDI-client proteins or any other work examining the direct effect of Ero1 on the folding of small peptide substrates. While our initial observations on the direct effect of Ero1 on conotoxin folding is intriguing, it may not be relevant in vivo and requires further testing using other small, cysteine-rich peptides.

It should be noted that the two conotoxin folding substrates used in this study only represent a small fraction of the structural biodiversity reported in cone snail venoms. Furthermore, these peptides originated from two different species of cone snail and the efficiency of enzymes sequenced from *C. geographus* may be lower for a peptide isolated from another species (i.e., μ-SmIIIA from *C. stercusmuscarum*). Ero1-assisted folding studies of a broader range of peptides with diverse structural scaffolds are likely to shed light on potential species- and peptide-specific folding kinetics.

Here, we present the discovery and initial functional characterization of the first Ero1 enzyme from the venom gland of cone snails, animals known for their high throughput production of small, disulfide-rich peptide toxins. Future in-depth characterization of different isoforms of Ero1 from cone snails and their contribution to the folding of a wider array of peptide toxins is likely to expand our understanding of the molecular adaptations evolved to facilitate the production of small, structurally diverse peptides in cone snails and other venomous animals.

## 4. Materials and Methods

### 4.1. Specimen Collection

Specimens of *Conus geographus* were collected from Sogod Island in the Central Philippines. The venom gland was dissected and stored in RNAlater^®^ (Thermo Fisher Scientific, Waltham, MA, USA) at −80 °C until further processing.

### 4.2. Sequencing and Cloning of Ero1

Total RNA was extracted from the venom gland using the RNAEasy MiniKit (Qiagen, Hilden, Germany). cDNA was synthesized using SMART MMLV reverse transcriptase (Clontech, Palo Alto, CA, USA) with a 1:1 mixture of oligo dT and random hexamer primers. Oligonucleotides for amplifying cone snail Ero1 were designed based on a sequence obtained from the venom gland transcriptome of *Conus bullatus* [33]. PCR was performed using Advantage 2 DNA polymerase (Clontech) (Sense: 5′-CGATGTTACAGTTGTCTCTG-3′; Antisense: 5′-CACAGGTGCTGCTCCAAA-3′). The PCR amplicon was gel-purified, ligated into the pGEM-T Easy vector (Promega, Madison, WI, USA) and transformed into *E. coli* DH10B cells (Novagen, Madison, WI, USA). Bacteria containing the Ero1-encoding plasmid were grown overnight in luria broth (LB) containing 100 µg/mL ampicillin, and DNA was extracted using the Qiagen miniprep kit. Plasmids isolated from overnight cultures were sequenced at the University of Utah Sequencing Core Facility. The Ero1 open reading frame (ORF) lacking the N-terminal signal peptide was amplified from pGEM-T Easy vectors using Advantage 2 DNA polymerase (Clontech) with oligonucleotides containing BamH1 and Sal1 restriction sites (Sense: 5′-CACACAGGATCCAGTGACAATGTTTCTCAAGGCTG-3′; Antisense: 5′-CACACAGTCGACTCGTCATCTCAGCAAGGCACG-3′). PCR amplicons were cut and inserted into the pGEX-6P-2 vector containing an N-terminal glutathione S-transferase (GST) tag separated from the insert by a PreScission Protease cleavage site.

### 4.3. Expression and Purification of Ero1

The GST–Ero1 fusion construct was expressed in Origami B DE3 competent cells (Novagen). Overnight cultures were grown in LB medium containing 100 µg/mL ampicillin, 50 µg/mL kanamycin, and 10 µg/mL tetracycline until the optical density (OD) at 600 nm was 0.6. Cells were induced by adding isopropyl-β-d-thiogalactopyranoside (IPTG) to a final concentration of 40 µM. Cultures were shaken at 25 °C for 20 h. Cells were lysed by probe sonication in lysis buffer (LBF: 50 mM Tris, 150 mM NaCl, 1× SigmaFast protease inhibitor tablet (Sigma-Aldrich, St. Louis, MO, USA), pH 7.5), followed by centrifugation at 20,000× *g* for 25 min to remove cellular debris. Protein lysates were run twice over a column containing Pierce Glutathione Agarose (Thermo Fisher Scientific) equilibrated with LBF. Bound protein was washed with LBF and eluted with LBF containing 10 mM reduced glutathione (GSH), adjusted to pH 8. GST–Ero1 fusion constructs were dialyzed against LBF using Spectra/Por 1000 molecular weight cut-off dialysis tubing (Spectrum Labs) with two buffer exchanges to remove glutathione. Cleavage of the GST tag from the Ero1 protein was achieved by incubating with a 1:4 ratio of PreScission Protease to Ero1 for 4–12 h at 4 °C. Following incubation, the mixture was applied to a column containing Pierce Glutathione Agarose as described above. Ero1 collected in the eluent was applied to a Superdex 75 size exclusion chromatography (SEC) column (Pharmacia, Pharmacia LKB Biotechnology AB, Uppsala, Sweden) and eluted in 50 mM Tris-HCl, 150 mM NaCl, pH 7.5. Fractions were analyzed using reducing (50 mM DTT) and non-reducing SDS-PAGE. Fractions containing the Ero1 monomer were combined and applied to a column containing Pierce Glutathione Agarose to remove remaining GST. Protein concentrations were determined using the proteins’ molar extinction coefficient (51,340 cm^−1^ M^−1^). Purified Ero1 lacks the N-terminal signal sequence but has an additional N-terminal pentapeptide with the sequence GPLGS.

To fully oxidize *Conus* Ero1, an aliquot of purified enzyme was incubated with 20 mM potassium ferricyanide for 1 h on ice. Complete reduction was achieved by incubation with 100 mM DTT prior to analysis by SDS-PAGE.

### 4.4. Cloning, Expression and Purification of C. Geographus PDI and csPDI

*Conus geographus* PDI (GenBank: AMM62646) and csPDI (GenBank: AMM62654) were expressed, purified and quantified as previously described [2].

### 4.5. Oxygen Consumption Assay

Oxygen consumption was measured at 30 °C using a FireStingO_2_ fiber-optic oxygen meter (Pyro Science GmbH, Aachen, Germany) by mixing 2, 4 or 10 µM PDI or csPDI with 4 µM Ero1 in air-saturated buffer containing 50 mM Tris-HCl (pH 8.1), 150 mM NaCl and 10 mM GSH at 30 °C.

### 4.6. Redox State Analysis of Conus PDI or csPDI in the Presence of Ero1

*Conus* PDI or csPDI was reduced with 10 mM DTT for 10 min at 4 °C, and DTT was removed using a PD-10 desalting column (GE Healthcare, Chicago, IL, USA). Reduced PDI was incubated with *Conus* Ero1 in air-saturated buffer containing 50 mM Tris/HCl (pH 7.5) and 300 mM NaCl at 30 °C. At the indicated time points, the reaction was quenched with 1 mM maleimide-PEG-2k [32]. All samples were separated by non-reducing SDS-PAGE followed by staining with Coomassie Brilliant Blue [12].

### 4.7. Synthesis of Peptide Substrates for Oxidative Folding Studies

Two model conotoxins were selected for oxidative folding studies because of their well-characterized folding properties [19,20] and their previous use as a folding substrate for *Conus* PDI and csPDI. ω-GVIA is an N-type calcium channel blocker originally isolated from the venom of *C. geographus* [34] and μ-SmIIIA is a sodium channel inhibitor isolated from the venom of *C. stercusmuscarum* [35]. Peptides were synthesized at the peptide synthesis core facility at the University of Utah, USA on a solid support by an automated peptide synthesizer (Applied Biosystems 431 Peptide Synthesizer) using Fmoc (N-(9-fluorenyl) methoxycarbonyl) protected amino acids, HBTU (O-(benzotriazol-1-yl)-1,1,3,3-tetramethyluronium hexafluorophosphate), and diisopropylethylamine.

Peptides were cleaved from the resin by treatment with reagent K (trifluoroacetic acid (TFA)/thioanisole/ethanedithiol/water/phenol (82.5/5/2.5/5/5 by volume)) for 3.5 and 2.5 h for ω-GVIA and μ-SmIIIA, respectively. The peptides were subsequently filtered, precipitated and washed with cold methyl *tert*-butyl ether. Linear peptides were purified by reversed-phase high performance liquid chromatography (RP-HPLC) on a semi-preparative C_18_ column (Vydac, 5µm particle size, 10 mm × 250 mm, Grace) using a linear gradient from 5–50% buffer B (90% acetonitrile (ACN)/0.1% trifluoroacetic acid (TFA)) over 45 min. Buffer A was 0.1% TFA/water. Absorbance was monitored at 220 and 280 nm. Concentrations were determined spectrophotometrically using the peptides’ molar absorption coefficient at 280 nm (without disulfide bonds). The purity and correct molecular mass of the final synthetic material were verified by RP-HPLC and mass spectrometry. Correctly folded peptide standards were obtained from the peptide synthesis facility at the Salk Institute, CA, USA.

### 4.8. Oxidative Folding Assays

Folding assays using recombinant *Conus* PDI, *Conus* csPDI (expressed and purified as previously described [2]) and *Conus* Ero1 were performed in aerated folding buffer (75 mM Tris-HCl, 1 mM EDTA, 0.2 mM FAD) containing (a) no enzyme, (b) 2 µM of PDI or csPDI and (c) 2 µM of PDI or csPDI and 4 µM Ero1.

To investigate potential synergistic effects, folding reactions were performed in the presence of (a) Ero1 and PDI, (b) Ero1 and csPDI, and (c) Ero1 and PDI in combination with csPDI. In these experiments, Ero1 was used at a concentration of 4 µM, and PDI and csPDI were tested at either 2 or 10 µM.

All reactions were incubated at room temperature for 20 min and initiated by adding 20 µM reduced, synthetic conotoxin substrate (ω-GVIA or μ-SmIIIA). Reactions were quenched at different time points by adding formic acid (10% final) and analyzed by RP-HPLC as described previously [2]. Briefly, native peptides were distinguished from linear forms based on their characteristic elution profiles [19,20], by comparing the elution profiles to native standard peptides and by mass spectrometric (MS) analysis of manually collected reversed-phase fractions (MALDI-TOF mass spectrometer, positive reflector mode, Voyager, AB SCIEX, Applied Biosystems, Foster City, CA, USA). To determine the amount of linear and native peptide, the area under the curve was calculated at each time point (*n* = 2 for each time point, mean ± SD).

### 4.9. Statistical Analysis

For oxygen consumption assays (Figure 3) and redox state analysis (Figure 4), statistical analysis calculated by one-way or two-way analysis of variance (ANOVA) with Tukey HSD (honestly significance difference) post hoc testing. All statistical tests were performed using KaleidaGraph software (Synergy Software, Reading, PA, USA) at a significance level of α = 0.05. For oxidative folding studies, early and late time points of folding (Figure 5) were analyzed using two-tailed Student’s *t*-tests with unequal variance (Welch’s correction) using GraphPad Prism software (version 7). For statistical analysis of different time points of folding (Figure 6), data was analyzed using two-way ANOVA with time and treatment as independent variables using GraphPad Prism software (version 7).

## Supplementary Materials

The following are available online at http://www.mdpi.com/1422-0067/19/11/3418/s1.

## Abbreviations

ACN	acetonitrile
csPDI	conotoxin-specific protein disulfide isomerase
DTT	dithiothreitol
ER	endoplasmic reticulum
Ero1	endoplasmic reticulum oxidoreductin-1
FAD	flavin adenine dinucleotide
Fmoc	N-(9-fluorenyl)methoxycarbonyl
HBTU	O-(benzotriazol-1-yl)-1,1,3,3-tetramethyluronium hexafluorophosphate
ORF	open reading frame
PDI	protein disulfide isomerase
RP-HPLC	reversed-phase high performance liquid chromatography
SEC	size exclusion chromatography
TFA	trifluoroacetic acid
