# Supplementary material for: Ero1-Mediated Reoxidation of Protein Disulfide Isomerase Accelerates the Folding of Cone Snail Toxins
Source: Int J Mol Sci. 2018 Oct 31;19(11):3418. doi: 10.3390/ijms19113418 (PMC6275033; doi:10.3390/ijms19113418)
Supplement: Supplementary file 1 [file ijms-19-03418-s001.pdf]

Appendix

Supporting Tables 1 and 2: Statistical analyses of oxygen consumption assays (Figure 3) and oxidative folding experiments (Figure 5).

Supp. Table 1: Data for Figure 3, Data for Statistical Analysis for Figure; Tables show *P* values of two-tailed Student's t-tests with unequal variance (Welch's correction) using KaleidaGraph statistical software (Synergy Software)

Ero1:PDIs = 4 μM:2 μM, oxygen consumption by Ero1

Reaction time	5 min		10 min		30 min	
	only Ero1	PDI+Ero1	only Ero1	PDI+Ero1	only Ero1	PDI+Ero1
PDI+Ero1	0.0203		0.0144		0.0003	
csPDI+Ero1	0.1000	0.1802	0.1224	0.0226	0.0714	0.0092

Ero1:PDIs = 4 μM:4 μM, oxygen consumption by Ero1

Reaction time	5 min		10 min		30 min	
	only Ero1	PDI+Ero1	only Ero1	PDI+Ero1	only Ero1	PDI+Ero1
PDI+Ero1	0.0027		0.0015		<0.0001	
csPDI+Ero1	0.0180	0.0019	0.0116	<0.0001	0.0110	<0.0001

Ero1:PDIs = 4 μM:10 μM, oxygen consumption by Ero1

Reaction time	5 min		10 min		30 min	
	only Ero1	PDI+Ero1	only Ero1	PDI+Ero1	only Ero1	PDI+Ero1
PDI+Ero1	0.0002		<0.0001		0.0017	
csPDI+Ero1	0.0163	<0.0001	0.0240	<0.0001	0.0081	0.0002

Supp. Table 2: Data for Statistical Analysis for Figure; Tables show *P* values of two-tailed Student's t-tests with unequal variance (Welch's correction) using GraphPad Prism software (version 7).

Panel A, left: GVIA, 20 min, disappearance of linear form

	no enzyme	Ero1	PDI	csPDI	PDI+Ero1
Ero1	0.0048				
PDI	0.0578	0.0031			
csPDI	0.0143	0.0018	0.0217		
PDI+Ero1	0.0031	0.0057	0.002	0.002	
csPDI+Ero1	0.0026	0.0031	0.0016	0.0013	0.0567

Panel A, right: GVIA, 100 min, appearance of native form

	no enzyme	Ero1	PDI	csPDI	PDI+Ero1
Ero1	0.0038				
PDI	0.0034	0.0188			
csPDI	0.0014	0.0055	0.0023		
PDI+Ero1	0.0001	0.0004	<0.0001	0.0023	
csPDI+Ero1	<0.0001	0.0006	<0.0001	0.0006	0.0003

Panel B, left: SmIIIA, 16 min, disappearance of linear form

	no enzyme	Ero1	PDI	csPDI	PDI+Ero1
Ero1	0.0046				
PDI	0.0032	0.0121			

csPDI	0.0032	0.0132	0.256		
PDI+Ero1	0.0002	0.0047	<0.0001	0.0001	
csPDI+Ero1	0.0002	0.0036	0.0002	0.0002	0.0182

Panel B, right: SmIIIA, 64 min, appearance of native form

	no enzyme	Ero1	PDI	csPDI	PDI+Ero1
Ero1	N/A				
PDI	N/A	0.0004			
csPDI	N/A	0.0004	N/A		
PDI+Ero1	N/A	0.0239	N/A	N/A	
csPDI+Ero1	N/A	0.0026	N/A	N/A	0.0274

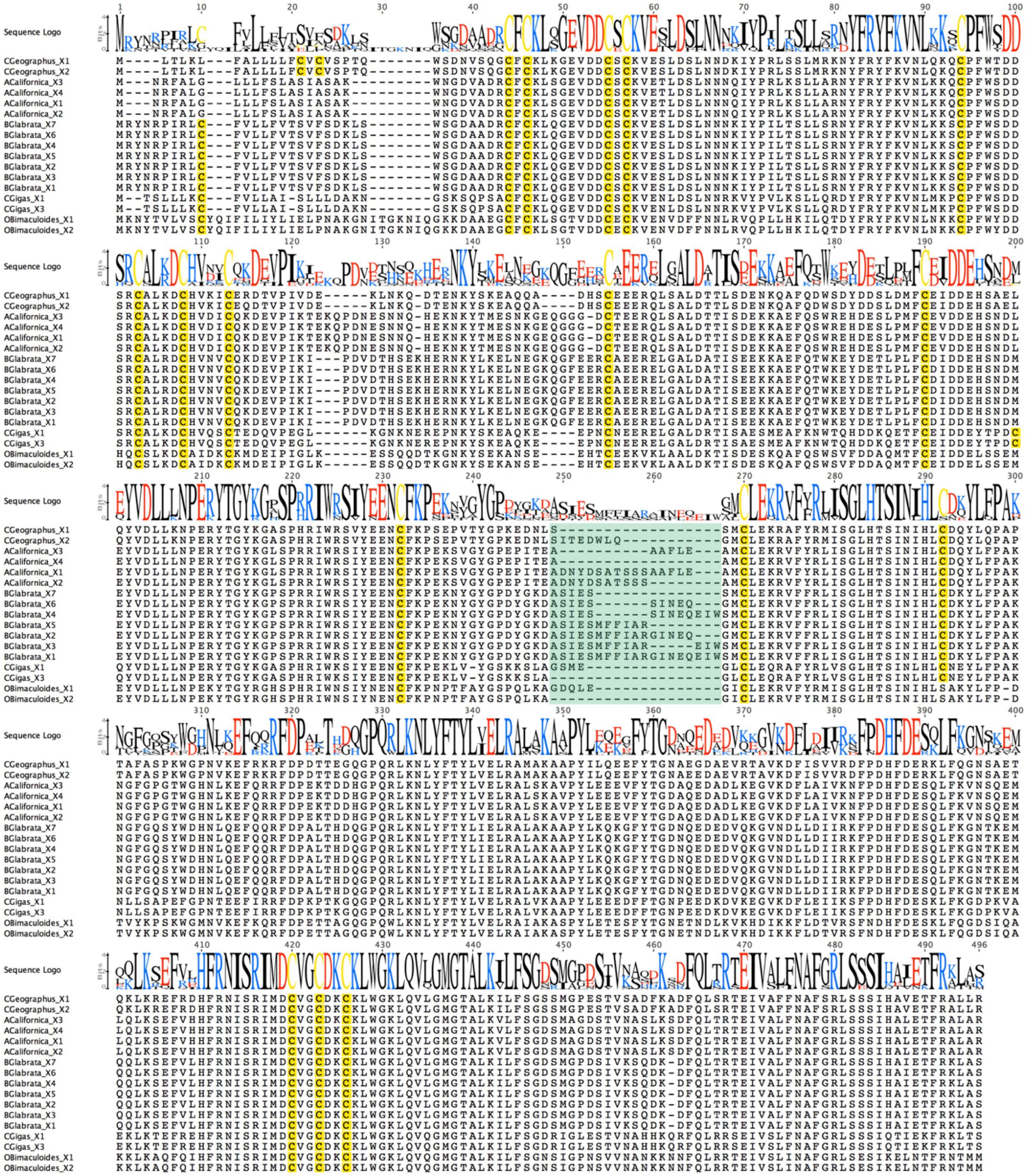

Supporting Fig. 1. Alignment of *Conus geographus* Ero1 X1 and X2 with that of other molluscan Ero1 isoforms shows high variability in a distinct region of the enzyme (green box). Cysteines are colored yellow. Alignment and sequence logos were created using Geneious software (version 8.1.3).

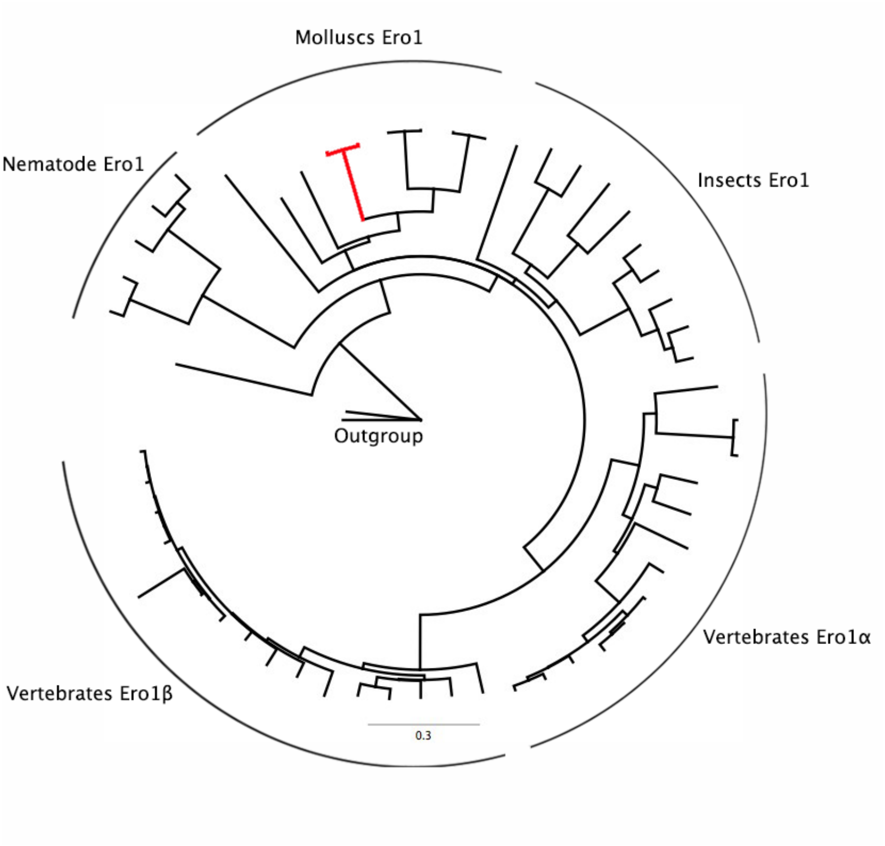

Supporting Fig. 2. Phylogenetic tree of Ero1 sequences from *Conus geographus* (red lines) and other Ero1 enzymes retrieved from the NCBI protein database. *C. geographus* Ero1 isoforms group with other molluscan Ero1 sequences. In vertebrates Ero1 enzymes can be classified into Ero1*α* and Ero1β. This distinction is absent in invertebrates (including *C. geographus*) and most likely occurred due to a gene duplication event in an ancestral vertebrate species. Neighbor-joining tree was generated in Geneious (version 8.1.3) using the Juke-Cantor genetic distance model. Two tapeworm species served as outgroup (*Echinococcus granulosus* and *Hymenolepis microstoma*).
